# Advances in ocular motor and pupil biomarkers for neurological disorders

**DOI:** 10.1093/braincomms/fcag102

**Published:** 2026-03-20

**Authors:** Ana Coito, Dominik Brügger, Tatiana Brémovà-Ertl, Pia Massatsch, Mathias Abegg, Konrad P Weber, Anke Salmen

**Affiliations:** machineMD, Bern 3008, Switzerland; machineMD, Bern 3008, Switzerland; Department of Neurology, University Hospital Bern, Bern 3008, Switzerland; machineMD, Bern 3008, Switzerland; machineMD, Bern 3008, Switzerland; Department of Ophthalmology, University Hospital Zurich, Zurich 8091, Switzerland; Department of Ophthalmology, University Hospital Zurich, Zurich 8091, Switzerland; Department of Neurology, University Hospital Zurich, Zurich 8091, Switzerland; Department of Neurology, St.Josef-Hospital, Ruhr-University Bochum, Bochum, Germany

**Keywords:** neurological disorders, ocular motor biomarkers, oculomics, pupillometry, virtual reality

## Abstract

Neurological disorders are often difficult to diagnose and monitor, particularly in the early stages when symptoms may be subtle or nonspecific. Because the visual system engages a large portion of the cerebral cortex and relies on well-defined neural pathways, it offers a unique and accessible window into brain function. In this context, the concepts of oculomics and oculometrics have gained increasing attention. Oculomics refers to the study of systemic and neurological diseases through ocular biomarkers, while oculometrics involves the computational quantification of eye and pupil parameters. Together, these approaches provide noninvasive, objective, and reproducible methods to assess neurological function, with strong potential to improve diagnostic precision, monitor disease progression, and guide individualized care.

This review synthesizes recent advances in ocular motor and pupillary biomarkers in three major neurological conditions: multiple sclerosis, Parkinson’s disease, and Alzheimer’s disease. In multiple sclerosis, early ocular motor disturbances such as internuclear ophthalmoplegia, saccadic dysmetria, and impaired smooth pursuit are frequently observed and may reflect brainstem and cerebellar involvement. Relative afferent pupillary defect, objectively measured with pupillometry, is a strong indicator of optic neuritis. In Parkinson’s disease, impaired saccadic initiation, hypometric saccades, and convergence abnormalities reflect basal ganglia dysfunction, while pupil irregularities suggest underlying autonomic imbalance. In Alzheimer’s disease, impairments in saccades, smooth pursuit, fixation instability, and the pupillary light reflex have been associated with early cortical and brainstem pathology, reflecting deficits in attention, executive control, and cholinergic function.

We also discuss the integration of eye-tracking data with neuroimaging and electrophysiology biomarkers to support multimodal diagnostic frameworks with the potential to improve diagnostic accuracy and disease monitoring. In addition, we highlight how recent technological developments in virtual reality-based eye-tracking could offer immersive, standardized testing conditions to enable scalable implementation of oculometric assessments in clinical practice.

As the fields of oculomics and oculometrics continue to evolve, these approaches hold promise to bridge the gap between research and clinical application. However, large-scale validation studies, standardized protocols, and normative datasets are essential for broader clinical adoption. By embedding ocular motor and pupillary biomarkers into routine neurological assessments, clinicians may be able to detect disease earlier, differentiate between overlapping syndromes, and monitor therapeutic outcomes more effectively.

## Teaser

Coito *et al*. report that eye movement and pupil response measures offer sensitive and objective indicators of neurological disorders. They summarize evidence showing how these oculometric parameters reflect underlying impairment of brain function and conclude that such measures hold strong potential for early diagnosis, monitoring disease progression, and guiding clinical care.

## Graphical Abstract

**Graphical Abstract:**
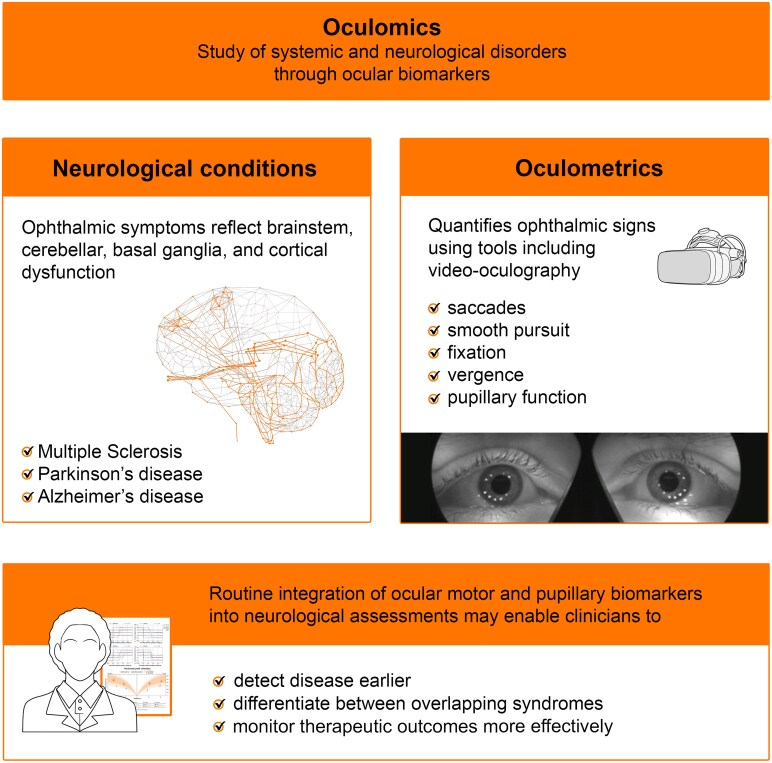

## Introduction

Neurological disorders may be challenging to diagnose and monitor, requiring precise, multimodal assessments to achieve early and accurate diagnosis. Early detection and effective monitoring are critical for improving outcomes; however, traditional clinical tools often fall short in sensitivity, especially in the early stages of disease progression.^1-4^

The neural circuitry of visual and ocular motor processing is known in such detail that an examination of eye and pupil responses allows for the localization of lesions in this circuitry.^5^ Given that the visual system occupies 20%–25% of the cerebral cortex,^6^ its assessment provides valuable insights into widespread brain function and dysfunction. Lesions in this system may be caused by neuroinflammatory and/or neurodegenerative conditions such as multiple sclerosis (MS),^7-9^ Parkinson’s disease (PD),^10-12^ Alzheimer’s disease (AD),^12-14^ and others. A neuro-ophthalmological evaluation, including recording and analysis of different eye and pupil movements, is a valuable tool to understand the functional integrity of brain structures and should be part of the neurological examination to detect manifestations that can ultimately help in the diagnosis of neurological conditions.^15,16^ However, in clinical practice, neuro-ophthalmic signs are frequently missed or misinterpreted, leading to diagnostic delay or error.^17^ For instance, studies have shown that many patients initially diagnosed with optic neuritis were later reclassified after specialist evaluation, underscoring the risk of misdiagnosis and delayed treatment.^18,19^

The measurement of eye and pupil movements with eye tracking technology can provide objective functional measures with reproducible results.^20^ Eye movements and pupil responses are increasingly being explored as digital biomarkers for neurological disorders.^21-25^ Within this context, the terms oculomics and oculometrics are gaining relevance.^23,26,27^ Oculomics, a term first coined in 2020 by Siegfried Wagner, Alaistar Denniston, Pearse Keane, and colleagues, refers to the study of systemic and neurological diseases through quantitative ocular measurements, such as eye movement dynamics, pupillary responses, and retinal structure.^28^ Oculometrics refers to the quantification and computational analysis of these ocular features, enabling high-resolution, reproducible, and objective assessments. Ocular motor and pupillometric parameters are key components of oculometrics. Together, the concepts of oculomics and oculometrics capture the potential of the eye as a window into brain function.

Recent developments in eye-tracking technology, including its integration into virtual reality (VR) headsets, enable the rapid, quantitative assessment of multiple ocular motor parameters in a single, streamlined examination.^29-32^ By automating measurements of visual fields, pupil reactivity, and eye movements (e.g. smooth pursuit, saccades, fixation, and vergence), these devices can facilitate early diagnosis, improve accessibility for patients, and enhance the accuracy of longitudinal monitoring. This quantitative approach to neuro-ophthalmic examination may represent an advancement over traditional clinical methods.

**Figure 1:**
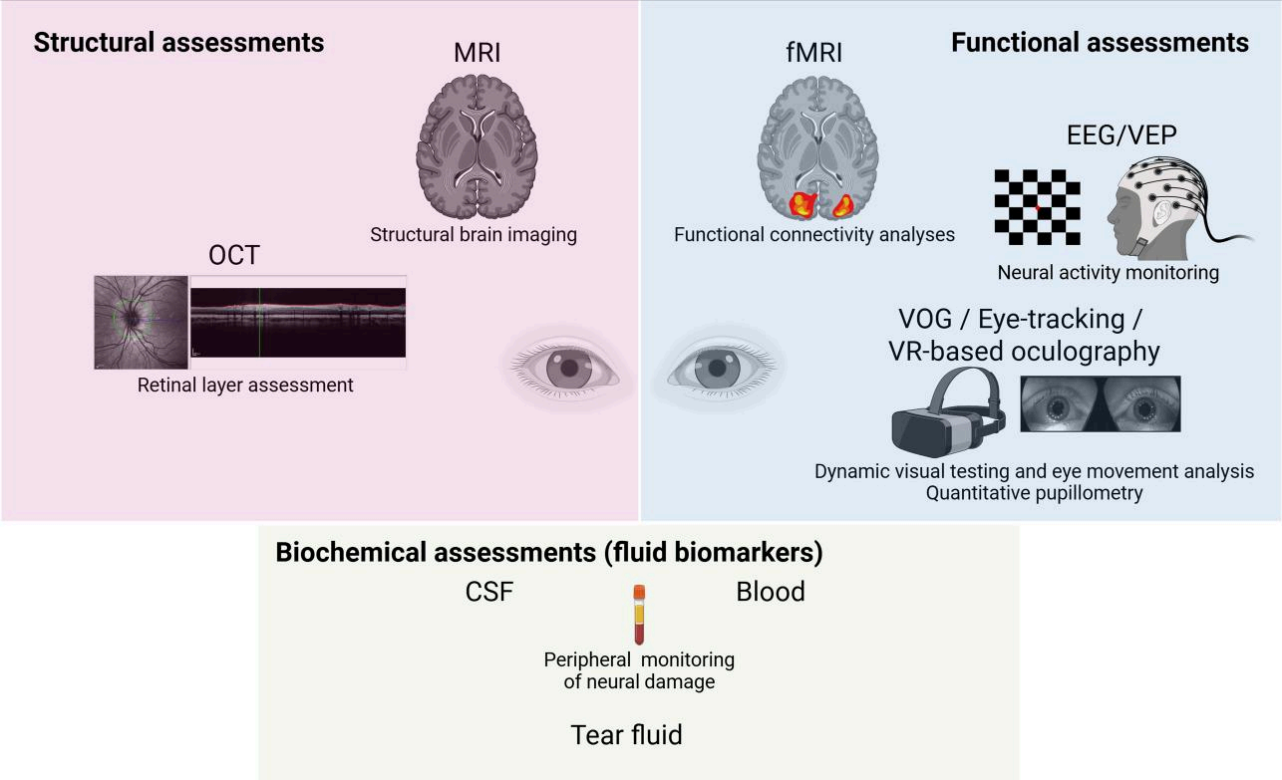
**Current main methods to assess brain structure, function, and neuronal integrity**. Understanding brain structure and function is essential for diagnosing and monitoring neurological conditions. Various methodologies provide complementary insights into neural integrity, dysfunction, and disease progression. Structural techniques (MRI and OCT) assess anatomical integrity; functional techniques (eye-tracking, quantitative pupillometry, EEG/VEP, and fMRI) measure dynamic neural responses; and biochemical fluid biomarkers (CSF, serum, plasma, and tear fluid) reflect underlying molecular and cellular processes. Together, these complementary modalities form the basis of oculomics and support a multimodal framework to evaluate the structural and functional correlates of neurological disorders. Created in BioRender. (2026) https://BioRender.com/nzyzoin.

The combination of eye-tracking technology with other modalities, including quantitative pupillometry (QP), neuroimaging techniques such as (functional) magnetic resonance imaging ((f)MRI), and optical coherence tomography (OCT), fluid biomarkers, and electrophysiology (electroencephalography (EEG), and visual evoked potentials (VEP)) may open new avenues for understanding the neural correlates of ocular motor function in neurological disorders. As illustrated in Fig. 1, structural and functional assessments provide complementary information, and their integration is increasingly important to capture the full spectrum of neurological dysfunction. This multimodal approach may allow researchers and clinicians to link specific eye movement patterns with underlying brain activity, providing a more comprehensive understanding of disease mechanisms and potential therapeutic targets.^33^ Advancements in machine learning and artificial intelligence (AI) have significantly improved the analysis and interpretation of eye-tracking data.^34-36^ These computational methods can detect subtle patterns and anomalies in eye movements that may not be apparent through traditional analysis techniques, potentially leading to earlier and more accurate diagnoses.^37^

This review summarizes the diagnostic and monitoring potential of ocular motor and pupillary biomarkers across some of the most common neurological conditions: MS, PD, and AD. We will discuss how these biomarkers may serve as indicators of neurological health and how, in combination with other biomarker modalities, they could contribute to a holistic assessment framework. By examining both established and emerging technologies, we provide a comprehensive overview and outlook of how advancements in this field may improve patient outcomes.

## Ocular motor and pupil biomarkers in neurological conditions and potential integration with other modalities

### Multiple sclerosis

#### Ocular motor and pupillary function impairments

MS, an autoimmune disease affecting the central nervous system, commonly results in ocular motor dysfunctions due to demyelinating lesions in brain regions critical for eye movement control. These disturbances are important components of oculomics, providing insights into disease mechanisms and informing both diagnosis and monitoring.

Eye movement disturbances are present in about 40%–76% of people with MS (PwMS) and often present early in the course of the disease.^7^ Eye movement impairments are common in all MS phenotypes, even at the earliest stages but are more prominent in progressive phenotypes.^38^ They have been proposed as biomarkers of early cognitive deficit and may help assess disease status and progression, as well as serve as a functional outcome to test novel therapeutic agents for MS.^8^

Among PwMS who present abnormal eye movements, the most frequently observed eye movement disorders are saccadic dysmetria (91%), internuclear ophthalmoplegia (INO) (68%), nystagmus (36%), vestibulo-ocular reflex (VOR) abnormalities (36%), impaired smooth pursuit (32%), misalignment of visual axes (32%), and impaired vergence (23%).^7,9,12,24,39-41^

**Figure 2:**
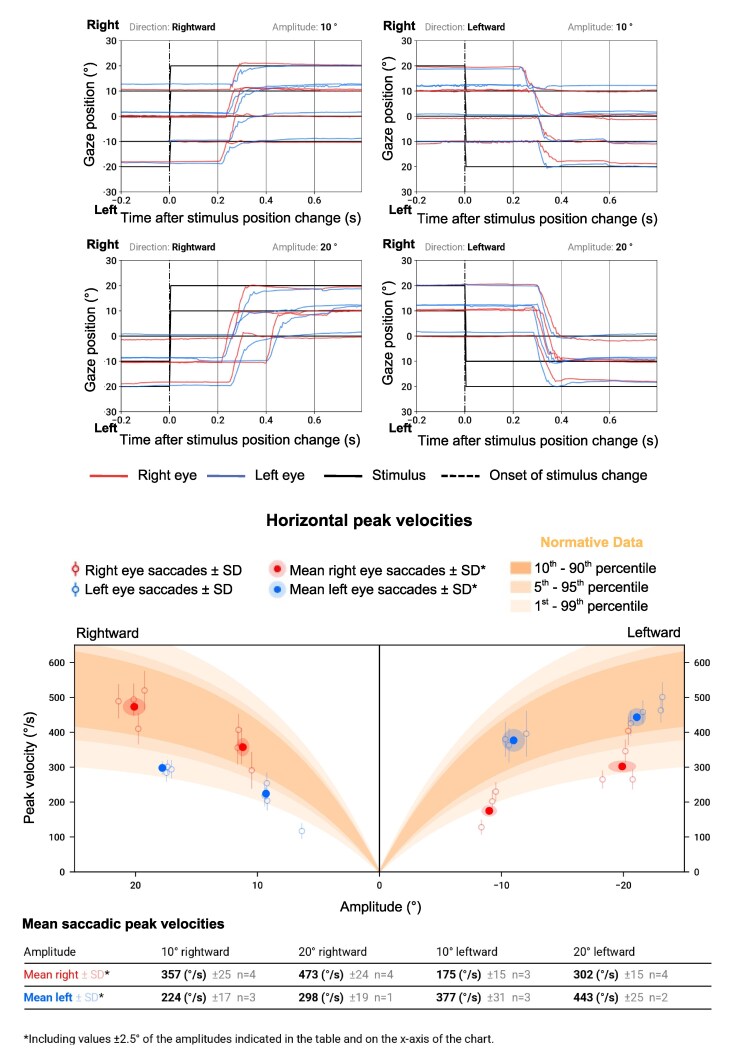
**Case example: horizontal saccades, measured using a VR-based VOG system, in a patient with clinically diagnosed relapsing-remitting MS.** The upper panels show horizontal saccades to 10° and 20° targets. The eye position traces (red/blue) follow these steps over time (x-axis). In a healthy subject, both eyes would make rapid, conjugate, step-like deflections closely aligned in time with the target jump. In this patient, the adducting eye is slower and hypometric compared with the abducting eye, producing disconjugate movements characteristic of bilateral INO. The lower horizontal main-sequence plots display peak velocity (y-axis) as a function of saccade amplitude (x-axis), with the shaded band indicating the normative range. Data points for the adducting eye fall clearly below the reference band due to markedly reduced peak velocities, while abducting-eye velocities lie closer to the normal range.

A hallmark ocular motor sign in MS is INO, which is characterized by slowed adduction in one eye and, in some cases, nystagmus in the abducting eye. INO is present in about a quarter of PwMS^42^ and reflects precisely localized lesions in the medial longitudinal fasciculus (MLF),^8^ a brainstem tract that integrates signals from the abducens and oculomotor nuclei to coordinate horizontal eye movements. The MLF also carries vestibular signals from the vestibular nuclei to the ocular motor nuclei, and thus, its disruption may impair the VOR. INO is highly specific for brainstem involvement and may precede structural abnormalities visible on conventional MRI.^38^ INO is associated with higher disability and worse cognition in PwMS.^42^ In some cases, slowed adducting saccades may be the only manifestation of INO.^9^ INO may serve as a marker of axonal and myelin integrity in MS.^8^ The use of oculometric tools, such as eye-tracking, enables objective detection and quantification of these subtle abnormalities. Patients with bilateral INO often also exhibit disrupted vertical eye movements, including impaired vertical gaze stability, VOR, optokinetic nystagmus (OKN), and smooth pursuit responses.^7,43^ Fig. 2 shows an example of INO as detected by a medical eye-tracker.

Because of their close connection with ocular motor nerve impairment, prosaccades are particularly valuable for assessing ocular motor function in PwMS, especially when measured with eye-tracking.^8,24,39^ Compared with healthy individuals, PwMS exhibit increased saccadic latency, reduced peak velocity and amplitude, and decreased saccadic accuracy.^44,45^ These parameters correlate with cognitive and functional decline^8^ and can be readily quantified using oculometric tools.

Smooth pursuit may also be an early disease marker.^24^ A study in clinically isolated syndrome (CIS) and PwMS showed that smooth pursuit integrity is substantially affected in both conditions, suggesting that low pursuit gain and increased saccadic amplitudes may be markers of disseminated pathology in CIS and MS.^46^ To note that accurate assessment of smooth pursuit requires intact foveal vision and the ability to maintain fixation, which are prerequisites for reliable interpretation of pursuit performance.

The number of fixational microsaccades in a 10-second recording was higher in PwMS and showed a significant association with higher EDSS, and worse performance in the nine-hole peg test, Symbol Digit Modalities Test (SMDT), and the Functional Systems Scores (FSS) for brainstem, cerebellar, and pyramidal involvement, suggesting that fixational microsaccades could be a proxy of MS disability and progression.^47^

PwMS with abnormal eye movements assessed clinically were more disabled than those with normal eye movements in 50 individuals with similar age and disease duration.^48^ A 2-year follow-up study corroborated the results with those who had abnormal eye movements remaining significantly more disabled (median EDSS of 7.0) than those with normal eye movements (median EDSS of 5.0).^49^ MRI scans proved detectable abnormalities in the brainstem or cerebellum of 60% of PwMS with abnormal eye movements and only 28% of PwMS with normal eye movements. The eye movement disorders most commonly noted in these studies were saccadic dysmetria, INO, and nystagmus and reflect demyelinating involvement of brainstem and cerebellar ocular motor pathways.^49^ Presence of compensatory saccades are also associated with a higher MS-related disability.^50^ Ocular motor assessment is sensitive in detecting subtle cognitive changes that occur early in the disease and may be used to characterize disruption to wide-ranging networks that support cognitive function.^51^ Studies assessing ocular motor function by video-oculography (VOG) in PwMS were able to detect changes at a preclinical stage by determining subclinical eye movement impairments even in the absence of characteristic lesions visible on MRI.^38,52^ These findings suggest that VOG may complement neuroimaging in confirming early demyelinating processes and monitoring disease progression.

Optic neuritis (ON) is a common disease manifestation in MS: in 33% of new MS cases, ON is the initial symptom^53^; 50%–70% of PwMS will experience ON at some stage of the condition.^54^ ON typically presents with subacute unilateral vision loss.^7,54,55^ A relative afferent pupillary deficit (RAPD) is present in 96% of acute unilateral optic neuritis cases, making it a reliable indicator of optic nerve damage in this population and a critical clinical sign for ON.^18,56,57^ RAPD assessment has traditionally relied on the swinging flashlight test, but quantitative pupillometry is increasingly used to enhance objectivity and reproducibility.^58-60^

Other pupillary function impairments are common in MS, with at least one pupillometric parameter (pupillary light reflex (PLR) latency, contraction amplitude, afferent dysfunction, and efferent dysfunction) significantly impaired in 60% of PwMS.^61^ Another study using quantitative pupillometry found that the initial pupil diameter and contraction amplitude were also lower in PwMS compared with healthy participants, and correlated with EDSS score and RNFL thickness, suggesting that alterations in PLR responses were associated with neurologic disability and retinal axonal loss.^62^

The most frequently reported ocular motor and pupillary impairments in MS are summarized in Table 1.

**Table 1:** Ocular motor and pupil function impairments in MS, PD, and AD

	MS	PD	AD
Saccades	Saccadic dysmetria INO (slow adducting saccades) ↑ Saccadic latency ↓ Saccadic peak velocity	Hypometric saccades (especially in the vertical direction) ↑ Saccadic latency ↓ Saccadic peak velocity ↓ Antisaccade latency	↑ Saccadic latency (pro- and antisaccades) ↓ Saccadic accuracy (pro- and antisaccades) ↑ Directional errors (pro- and antisaccades) ↓ Corrected errors (antisaccades)
Smooth pursuit	Impaired smooth pursuit	Impaired smooth pursuit	↓ Smooth pursuit gain
Fixation/gaze holding	Fixation instability	↑ Square-wave jerks ↓ Fixation duration	↓ Fixation duration
Ocular alignment	Phoria/Tropia	not reported	not reported
Vergence	Impaired vergence	Convergence insufficiency	not reported
Pupil	RAPD due to optic neuritis ↓ Initial pupil diameter and contraction amplitude	↑ PLR latency ↓ Amplitude ↓ Constriction velocity and acceleration	↑ PLR latency ↓ Amplitude ↑ re-dilation velocity in response to light
Others	Impaired VOR Impaired OKN ↑ Involuntary microsaccades	not reported	not reported

AD: Alzheimer’s Disease; INO: Internuclear Ophthalmoplegia; MS: Multiple Sclerosis; OKN: Optokinetic Nystagmus; PD: Parkinson’s Disease; PLR: Pupillary Light Reflex; RAPD: Relative Afferent Pupillary Reflex; VOR: Vestibular-Ocular Reflex.

**Figure 3:**
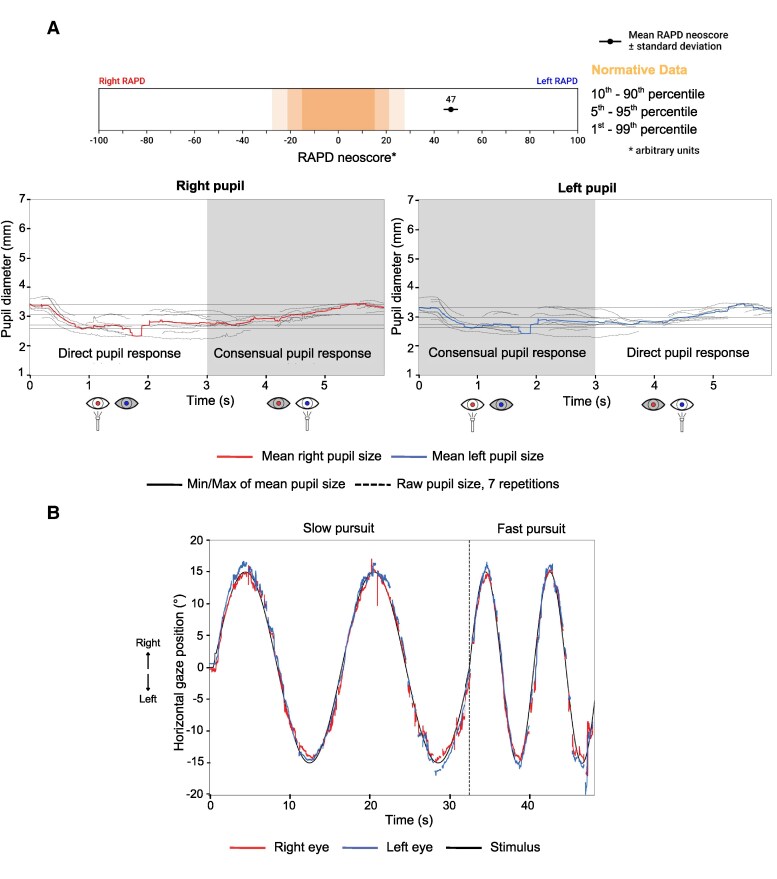
**Case example: afferent pupillary function and smooth pursuit, measured using a VR-based VOG system, in a patient with clinically diagnosed relapsing-remitting MS.** (**A**) Afferent pupillary function (swinging flashlight paradigm). The top bar summarizes the relative afferent pupillary defect (RAPD) estimate. The lower plots show pupil diameter (y-axis) over time (x-axis) for the right and left pupils during alternating monocular illumination. In a healthy response, pupil size is symmetric in light and darkness, and both pupils dilate similarly after darkening the screens, reflecting intact parasympathetic and sympathetic efferent pathways. In this patient, illumination of the left eye produces a much smaller and less sustained constriction in both pupils, followed by a pupillary escape, whereas illumination of the right eye elicits a larger, more sustained response. This asymmetry is consistent with a left RAPD, consistent with a history of left optic neuritis. (**B**) Horizontal smooth pursuit. Slow and fast horizontal smooth-pursuit traces are plotted alongside the sinusoidal target trajectory. In a healthy individual, eye traces would closely overlap the target, with minimal lag and few corrective saccades. Here, pursuit gain is reduced: the tracking is interspersed with catch-up saccades, giving the trace a jerky appearance.

In Figs 2 and 3, we present a case example referring to the ocular motor and pupillary function of a 58-year-old woman with clinically diagnosed relapsing-remitting MS (RRMS) measured using a VR-based VOG system. In this patient, the peak velocity of the adducting eye is markedly slower than the velocity of the abducting eye on both sides (Fig. 2, bottom panel), characteristic of bilateral INO. As a result of previously left optic neuritis, a left RAPD can be seen in the afferent pupillary function plot (Fig. 3A). This manifests with a smaller constriction amplitude after illumination of the left eye as compared to illumination of the right eye. The small constriction is followed by a pupillary escape. The RAPD is visible with observation of either eye alone (Fig. 3A). The patient also presents jerky pursuit movements (Fig. 3B).

#### Potential integration with other modalities

MRI may miss small lesions in the MLF or other brain regions.^63,64^ VOG in PwMS is able to detect subclinical eye movement impairments even in the absence of visible MRI lesions.^38^ The integration of oculometric markers from VOG with neuroimaging has the potential to improve lesion detection and localization in PwMS.^52,63^ MRI assessments are specific but not as sensitive for detecting INO as VOG.^52^ These techniques should thus be complementary since MRI can identify the structural and VOG can quantify the functional deficit.^52^ Indeed, a study in 202 PwMS showed that MLF pathology was more clinically relevant and more frequently detected by infrared VOG than by MLF lesion rating on MRI.^65^ Combining diffusion tensor imaging (DTI)-based neuroimaging with eye movement measurements, namely with VOG, may be useful to characterize axonal integrity and myelin status and quantify tissue injury in the MLF, which may overcome the limited resolution of traditional MRI for infratentorial lesion detection.^8,66,67^

Visual-evoked potentials (VEPs), which measure cortical responses to visual stimuli, are a common electrophysiological technique used in ON. They can reveal delayed conduction times indicative of demyelination.^68-70^ They could be combined with ocular motor and pupillary assessments to provide a comprehensive functional evidence of visual pathway disruption in MS as well as to assess remyelination after therapy.

A study in 72 people with unilateral or asymmetric demyelinating optic neuropathy demonstrated that the presence and severity of RAPD in PwMS correlated with the degree of retinal nerve fibre layer (RNFL) loss as assessed by OCT.^71^ Thinning of the RNFL is indicative of axonal loss and is observed in PwMS.^72^ The degree of RNFL thinning has been associated with the severity of visual field loss and other functional impairments in PwMS.^73,74^ Pupillary function abnormalities could be paired with RNFL thickness to assess afferent visual pathway integrity.

### Parkinson’s disease

#### Ocular motor and pupillary function impairments

PD, a progressive neurodegenerative disorder, is marked by motor symptoms like bradykinesia, tremor, and rigidity, and a range of nonmotor symptoms. Ocular motor and pupillary signs are gaining attention as potential early indicators and disease progression markers in PD. Ocular motor impairments are highly prevalent in PD, with studies reporting abnormal eye movement patterns in approximately 75%–87% of individuals with PD.^75,76^ These dysfunctions may precede or follow motor symptoms.^77^ The substantia nigra pars reticulata is thought to modulate both saccades and smooth pursuit eye movements.^78^ Therefore, the degeneration of this region in PD results in both saccades and smooth pursuit impairments.^79^ Indeed, the most commonly reported ocular motor dysfunctions are impairments in convergence, saccades, smooth pursuit, and fixation.^75,76,80,81^ These parameters can be measured using oculometric tools, making them objective and reproducible candidates for monitoring disease progression.

Voluntary saccades are often hypometric in PD, in particular, in the vertical direction.^11,77,81-84^ Saccadic initiation delays, reduced peak velocity, and increased latency are common in PD, exceeding normal aging.^81,85^ Antisaccade latency may be a predictive marker of the 5-year onset of freezing of gait in PD.^86^ Studies report an abnormal convergence in about 70% of PD patients,^81^ often resulting in double vision.^87^ Fixation abnormalities, such as an increased frequency of small involuntary eye movements (square wave jerks), have been observed, indicating impaired gaze stability.^88^ A review of 26 studies reported that smooth pursuit impairments may be present in up to 67% of PD patients and have been linked to both severity of motor symptoms and disease progression in PD.^80^ Smooth pursuit eye movements seem to be affected in early PD as shown in a study where the smooth pursuit of people with early PD, who had not undergone any previous dopaminergic therapy, was assessed by electrooculography (EOG) before and after the administration of apomorphine.^89^

Pupil reactivity may also be affected in PD. Several studies analysing quantitative pupillometric parameters in PD have demonstrated that PLR latency is significantly increased, whereas amplitude, maximum constriction velocity, and maximum acceleration are decreased.^90-94^ A study in 132 PD patients found that pupillary constriction velocity decreased with PD progression, suggesting that parasympathetic pupillary dysfunction progresses with the progression of PD and that constriction velocity could be used to monitor PD progression.^95^ An autonomic imbalance in PD seems to be present in preclinical PD stages at least 5 years before diagnosis but could be present as long as 20 years before diagnosis.^96^ A study evaluating gaze stability, pupil size under stable light conditions, and eye movements during sustained fixation in 50 individuals with PD and 43 healthy participants found that shorter median pupil size and shorter fixation period were independently associated with diagnosis.^97^ Pupillometry may also be a sensitive tool for noninvasive evaluation of the peripheral effect of PD medication.^98^ It is an objective, fast, and cost-effective technique. Within the oculomics framework, pupillary abnormalities constitute promising non-motor biomarkers for early detection and longitudinal monitoring in PD.^99^

In most studies discussed above, participants with PD were taking dopaminergic medication. However, there is evidence that dopaminergic therapy can modulate ocular motor function, even in early disease. For instance, Bares *et al*. showed that dopaminergic treatment (apomorphine) improved smooth pursuit in early, drug-naive PD patients, indicating a dopaminergic influence on pursuit pathways.^89^ Another study found that levodopa improved the maximal oculomotor gaze range in all directions.^100^ Likewise, pupil reactivity measures may be influenced by PD medication.^94^

Key ocular motor and pupillary impairments reported in PD are summarized in Table 1.

**Figure 4:**
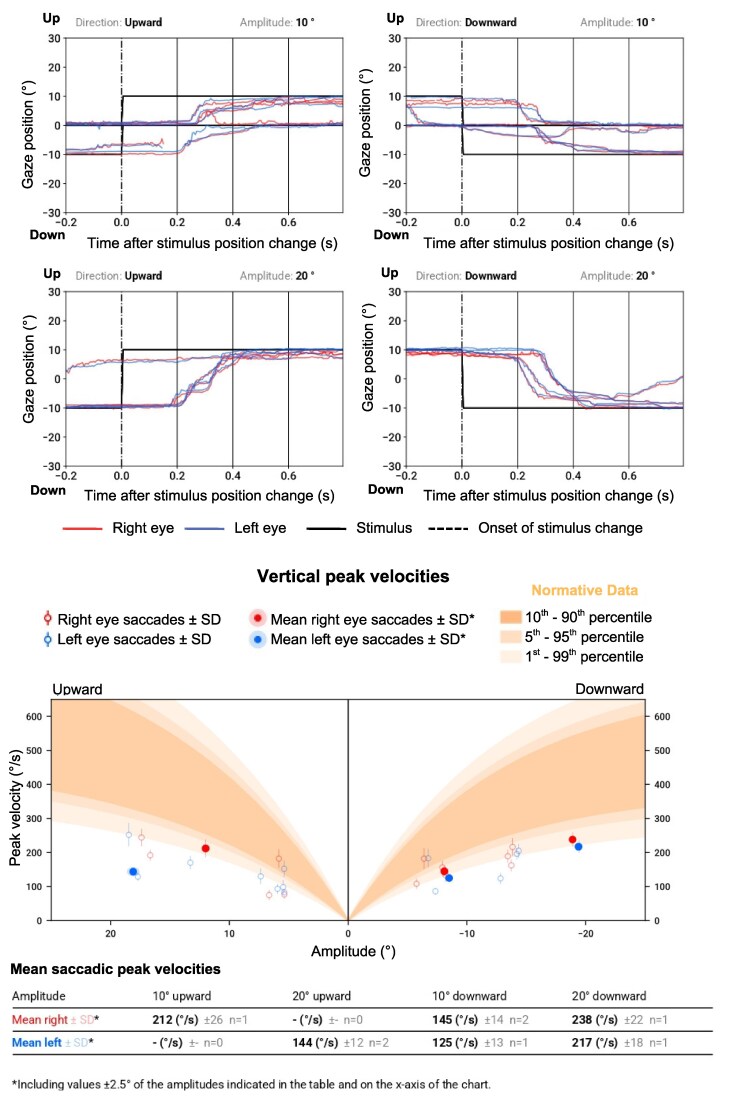
**Case example: vertical saccades, measured using a VR-based VOG system, in a patient with Parkinson’s disease.** The plots show upward and downward horizontal saccades following 10° and 20° stimulus steps. In a healthy subject, saccades would appear as rapid, sharply rising deflections tightly time-locked to target onset. Saccades frequently undershoot the target or take longer to reach it. Vertical saccades are markedly slow, with reduced peak velocities. The corresponding vertical main-sequence plots demonstrate that most data points lie below the normal velocity–amplitude band, which is consistent with reduced vertical saccadic velocities.

**Figure 5:**
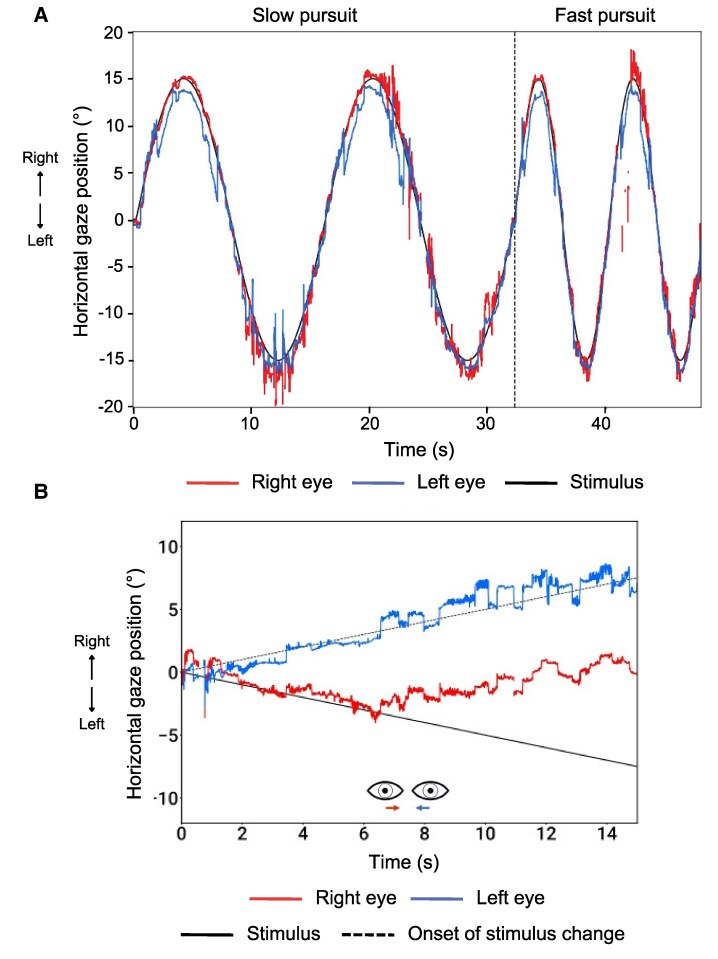
**Case example: horizontal smooth pursuit and convergence, measured using a VR-based VOG system, in a patient with Parkinson’s disease.** (**A**) Horizontal smooth Pursuit. Slow and fast horizontal smooth-pursuit traces are plotted alongside the sinusoidal target trajectory. In healthy pursuit, eye traces would closely follow the target with minimal lag. Here, both slow and fast pursuit show reduced pursuit gain, with the eyes lagging behind the target position throughout the cycle, especially in eccentric positions. Numerous catch-up saccades interrupt the traces, giving the trajectory a jerky, fragmented appearance. These abnormalities reflect impaired maintenance of continuous pursuit. (**B**) Convergence. The plots show the vergence response as gaze position during disparity-induced vergence stimuli moving over time. During a disparity-induced vergence task in which binocular disparity gradually increases, healthy individuals typically maintain symmetric convergence up to ≥10°. In this patient, convergence fails at approximately 5°, beyond which the two eye traces diverge rather than continuing to converge. This early breakdown of fusional convergence is consistent with convergence insufficiency.

A case example of ocular motor function of a patient with known PD (74-year-old, male, Hoehn & Yahr scale: 2) is presented in Figs 4 and 5. MDS-UPDRS part III was 23 points at the time of the eye tracking recording, indicating mild motor impairments. VOG recording showed a reduction in vertical saccadic velocity (Fig. 4). The recording also demonstrated a pathological jerky pursuit pattern (Fig. 5A). Image disparity induced by the convergence movement shows that fusional convergence is not maintained for an image disparity beyond approximately 5° (Fig. 5B), which is pathological as normal convergence should be sustained up to at least 10° of disparity.

#### Potential integration with other modalities

Imaging modalities have been used to search for PD biomarkers, such as conventional, iron sensitive, neuromelanin sensitive, diffusion sensitive, and resting state functional MRI, dopamine transporter imaging, positron emission tomography (PET, using the tracers [18F]fluorodopa or [11C]raclopride), and single photon emission computed tomography (SPECT).^101-109^ MR-based high-resolution morphometric and volumetric analyses of the substantia nigra have identified several promising imaging markers, such as reduced nigral volume, increased iron deposition, and alterations in nigrosome-1 integrity, which show potential as biomarkers for PD diagnosis and monitoring.^110,111^

Studies using structural MRI and DTI show that different structural impairments of grey (degeneration and atrophy of certain brain regions) and white matter correlate with ocular motor dysfunctions in PD.^112-114^ In a study in 36 PD, 30 PSP, 18 MSA, and 23 healthy participants, VOG was used to record smooth pursuit and saccades and DTI was used to record related microstructural alterations. It showed that in PSP, peak eye velocity was decreased compared to controls and correlated significantly with midbrain microstructural impairment.^113^ In MSA, smooth pursuit was disturbed by ‘catch-up’ saccades, and this was significantly correlated with decreased white matter in the middle cerebral peduncle. In PD, although the prevalence of saccadic intrusions was significantly increased, this was uncorrelated with cortical and subcortical white matter alterations. These findings suggested that eye movement impairments in PSP and MSA, but not in PD, are associated with diagnosis-specific regional white matter microstructural alterations.^113^ In an extended group of patients and healthy participants (39 PD, 32 PSP, 18 MSA, and 24 healthy), using structural MRI and VOG, ocular motor dysfunction measured by VOG was associated with cerebral atrophy in PD, pontocerebellar ocular motor structures atrophy in MSA, and midbrain atrophy in PSP as measured by MRI.^112^

A study in individuals with young-onset PD using fMRI showed that abnormal cortical activation during saccadic tasks may represent compensatory mechanisms for attentional and executive dysfunction.^115^ Altered functional connectivity, namely in regions within the default-mode network (DMN), has been observed in PD and linked to impaired eye movement control, specifically saccades, smooth pursuit, and presence of saccadic intrusions.^116-118^

Electrophysiological markers derived from EEG have also been explored as potential biomarkers for PD^119^ and are considered promising predictors of PD-related cognitive decline.^120^ Integrating eye-tracking data with EEG during cognitive tasks could potentially provide insights into PD-related cognitive impairments.

In addition, the combination of ocular motor and pupil biomarkers with clinical motor signs (bradykinesia, resting tremor, muscle rigidity) may improve PD detection and treatment response monitoring.^99^ Other objective motor measures, including gait and balance assessed via wearable sensors^121^ could also be integrated with ocular motor and pupil assessments to increase diagnostic sensitivity. To address this hypothesis, we are currently conducting a study in PD that evaluates both gait and ocular motor function (NCT06663826).

The integration of oculometrics with motor assessments, neuroimaging, and potentially biofluid biomarkers may support a more refined diagnostic and monitoring framework. Although such a multimodal oculomics approach is still emerging in PD, it holds promise for individualized disease profiling and therapy response tracking.

## Alzheimer’s disease

### Ocular motor and pupillary function impairments

AD is the most common cause of dementia and affects several brain areas responsible for higher cognitive functions and visual processing. Emerging evidence suggests that ocular motor and pupillary biomarkers can capture the cognitive and neurological decline characteristic of AD and mild cognitive impairment (MCI).^12-14,37,122-126^

Saccadic eye movement abnormalities are one of the most common ocular motor dysfunctions in AD.^127^ In eye tracking-based saccadic tasks, AD and MCI patients exhibit increased latency, lower accuracy, and higher directional error rates compared to healthy individuals.^13,122,123,128^ These impairments reflect disruptions in cortical regions responsible for executive function and visual attention, with studies showing correlations between saccadic dysfunction and cognitive deficits in AD. In a systematic review and meta-analysis of 26 studies, people with AD or MCI exhibited a significantly longer latency and lower accuracy rates in the prosaccade (SMD: −0.49 and 0.42, respectively) and antisaccade (SMD: −0.68 and 1.33, respectively) tasks, and lower corrected error rates (SMD: 1.32) in the antisaccade tasks.^129^ Another meta-analysis showed that people with AD or MCI had increased antisaccade error rates compared to controls in 14 of the 16 studies considered, with a pooled effect size of 1.13 (SE = 0.16, 95% CI: 0.93–1.34), indicating a large and consistent difference between patients and controls across studies.^130^ Studies suggest that prosaccades and antisaccade parameters, specifically saccadic errors, can differentiate AD from MCI, and these from cognitively normal individuals.^122,123,131^ Antisaccadic parameters could be used to assess executive function in individuals at risk for AD.^132^ AD patients have low smooth pursuit gain, with an increased number of corrective saccades interrupting pursuit movements.^12,127^ A study in three dementia types (AD, behavioural variant of frontotemporal dementia, and semantic variant of primary progressive aphasia) showed that distinct saccadic and pursuit patterns may help differentiate AD from other dementia types.^129,133^

Fixation abnormalities, such as shorter fixation durations and difficulty maintaining gaze on a target, have also been shown in AD. A prospective cohort study in 32 people with AD, 37 people with MCI, and 33 healthy participants, using an eye-tracker, showed that AD patients required longer periods to initiate fixation and frequently shifted their gaze involuntarily to distractors.^134^ Thus, these oculometric features may serve as digital proxies of attention and cognitive integrity.

Eye movement disorders are considered to be effective in tracking the severity and progression of AD.^135^

PLR metrics, as measured by quantitative pupillometry, offer additional diagnostic potential, particularly as they reflect early cholinergic dysfunction in the Edinger-Westphal nuclei, a brainstem area affected in early AD stages.^136,137^ AD patients demonstrate increased latency, reduced amplitude, and faster redilation phases in response to light stimuli, a pattern likely related to both cholinergic and noradrenergic dysfunctions.^136^ The locus coeruleus, which modulates sympathetic input to the eye, also shows neuronal loss in AD, contributing to altered baseline pupil size and abnormal PLR dynamics.^136,138^

The use of PLR as a measure of melanopsin retinal ganglion cell function could also be relevant for neurodegenerative disorders, where circadian dysfunction is present.^139^

These findings suggest that ocular motor and pupil biomarkers could serve as nonverbal indicators of cognitive impairment, complementing traditional assessments by capturing deficits in attention, executive control, and visuospatial processing, which are hallmarks of AD.

The main ocular motor and pupillary abnormalities described in AD are summarized in Table 1.

**Figure 6:**
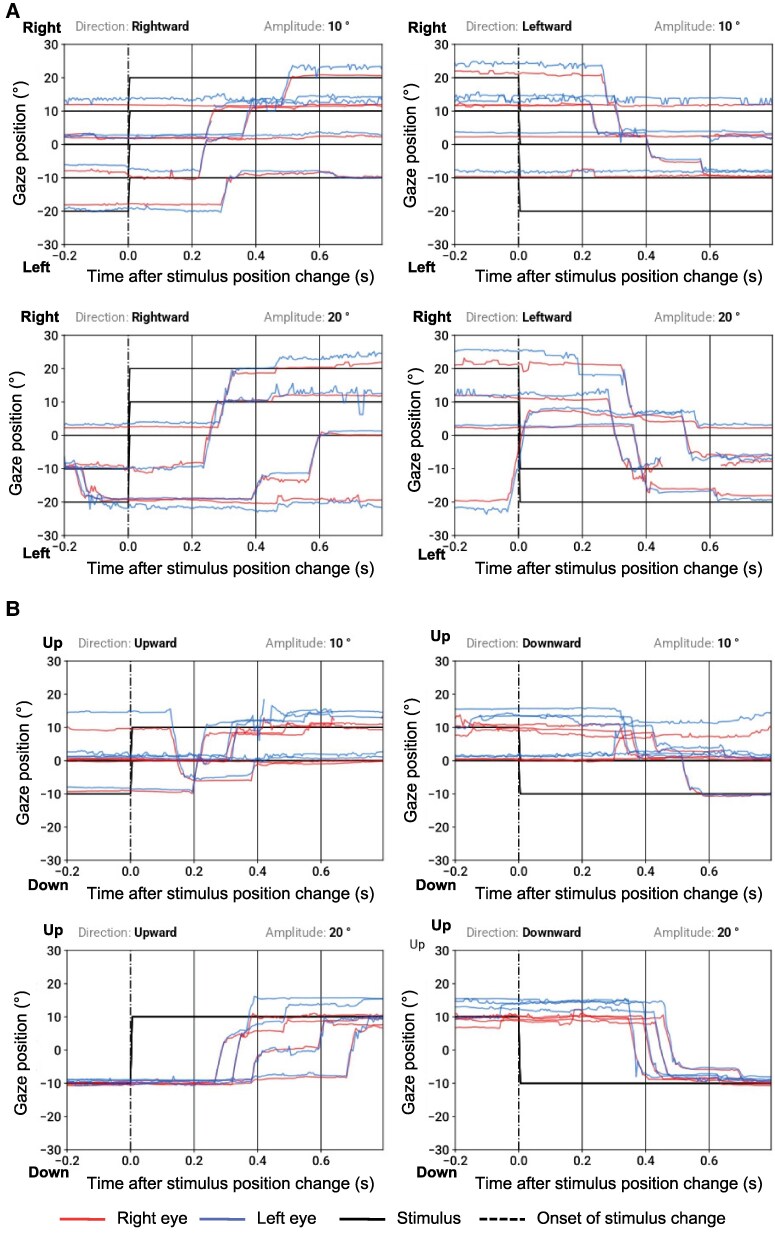
**Case example: horizontal and vertical saccades, measured using a VR-based VOG system, in a patient with early-stage Alzheimer’s disease.** (**A**) Horizontal Saccades. The plots display eye position over time following rightward and leftward 10° target steps. In this patient, horizontal saccades show marked trial-to-trial variability in latency, with some markedly delayed responses. (**B**) Vertical Saccades. For upward and downward target steps, eye movements again show high variability in latency. Some vertical saccades are delayed or slightly hypometric.

**Figure 7:**
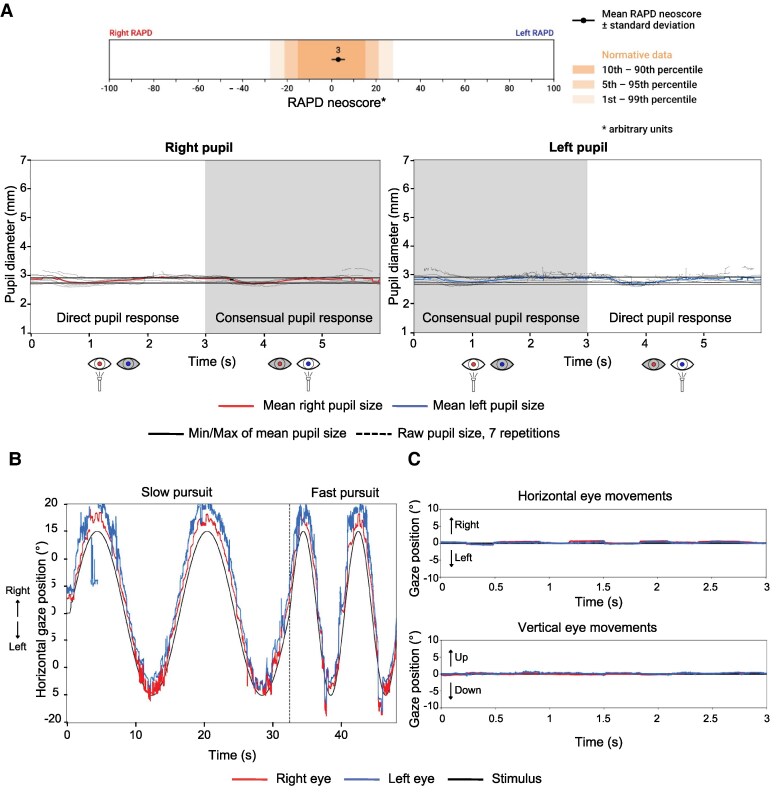
**Case example: afferent pupillary function, horizontal smooth pursuit, and fixation, measured using a VR-based VOG system, in a patient with early-stage Alzheimer’s disease.** (**A**) Afferent pupillary function. Pupil diameter over time is shown for alternating monocular illumination conditions (mimicking the swinging flashlight test). In this patient, both the right and left pupils exhibit small constriction amplitudes, minimal sustained constriction, and flattened response curves due to a blunted pupillary light reflex. (**B**) Horizontal smooth pursuit. Slow and fast horizontal smooth pursuit are plotted together with the sinusoidal target trajectory (black). In this patient, pursuit is jerky and fragmented, with clear phase lag and frequent catch-up saccades, especially at higher speeds. This reduced pursuit gain reflects impaired motion tracking and visuospatial integration, consistent with parietal and cortical dysfunction in early Alzheimer’s disease. (**C**) Binocular fixation in primary gaze. During steady fixation, healthy eyes remain stable with only minimal drift. In this patient, both horizontal and vertical traces show frequent small-amplitude saccadic intrusions, consistent with square-wave jerks.

Figures 6 and 7 show the ocular motor function of a 71- year-old woman with early-stage AD. The patient exhibits a high variability in saccadic latency (Fig. 6A and B), small pupillary amplitude (Fig. 7A), a jerky pursuit pattern (Fig. 7B), and fixation is interrupted by frequent small saccadic intrusions (square-wave jerks) (Fig. 7C).

#### Potential integration with other biomarkers

OCT has been widely used to assess retinal changes, which may serve as biomarkers for AD. Thinning of the RNFL, ganglion cell layer, and macular volume has been observed in AD patients and correlated with disease severity.^140-142^ The integration of OCT-derived measures with pupil or ocular motor metrics could provide insights into the functional consequences of structural alteration in the retina. Retinal amyloid-beta deposits, detectable via retinal hyperspectral imaging, have been proposed as potential AD biomarkers.^143^ Within an oculomics framework, combining retinal imaging with eye-tracking could potentially link amyloid burden to functional deficits as seen in pupil function, saccades, and smooth pursuit, further refining diagnostic accuracy.

Other ophthalmic parameters that have been studied include tear fluid composition, corneal nerve fibre length, choroidal thickness, abnormal protein deposits in the crystalline lens, optic nerve thickness, visual acuity, visual sensitivity, and stereopsis.^144^

Advanced neuroimaging techniques, including structural MRI, fMRI, and PET, have been instrumental in characterizing AD-related brain changes. Structural MRI studies often reveal hippocampal atrophy, cortical thinning, and volumetric reductions.^145^ PET imaging with tracers targeting amyloid and tau allows the assessment of the spatial pattern of tau deposition and its relation to amyloid-β pathology and neurodegeneration.^146^ This could potentially be combined with oculometric assessments to investigate how the pathological burden correlates with functional deficits in eye movements.

A systematic review and meta-analysis of functional connectivity studies in AD showed that the DMN is disrupted.^147^ A study in 125 individuals (40 with MCI, 43 with subjective cognitive decline (SCD), and 42 healthy participants) showed functional and structural alterations of the dorsal attention network (DAN) in MCI and SCD.^148^ Both the DMN and the DAN are critical for maintaining attention and executing goal-directed eye movements.^147,148^ Combining fMRI-based connectivity measures with ocular motor performance could elucidate the neural mechanisms underlying visuospatial and attentional impairments in AD.

EEG and magnetoencephalography (MEG) are used to study neural oscillations in AD, often revealing altered alpha, beta, and theta activity.^149^ By integrating EEG with eye-tracking metrics, the relationship between cortical dysfunction and ocular motor performance could be studied to identify unique neural signatures associated with visuospatial deficits in AD. Studies have combined eye-tracking with EEG recordings during visual or neuropsychological tasks.^150,151^ This combination has the potential to provide functional measures of attention and executive dysfunction in AD.

Several studies have attempted artificial intelligence (AI)-driven eye movement analysis, and the results hold promise for early and noninvasive AD diagnosis.^35-37,152^ Furthermore, multimodal frameworks combining electrophysiological, gait, speech, and cognitive parameters with eye movement parameters show potential for improving the detection of cognitive impairment, MCI, or AD.^153-157^ These integrative oculomics strategies may ultimately support precision medicine approaches for dementia care.

### Virtual reality and eye-tracking to help early detection of neurological disorders

VR has been increasingly applied in the medical field across various domains, including medical training,^158,159^ rehabilitation,^160^ medical diagnoses,^31^ mental health treatment,^161^ surgery planning,^162^ and patient education.^163^ In ophthalmology and neuro-ophthalmology, several VR-based devices have been applied to measure the visual field,^164^ RAPD,^60,165^ ocular alignment,^29,32,166^ and eye movements (gaze holding/fixation, saccades, and smooth pursuit).^29-31,167^

Eye-tracking technology noninvasively records precise metrics of eye movements, providing objective biomarkers of neurological dysfunction. These parameters, central to oculometrics, have shown utility in facilitating differential diagnosis among diseases with overlapping clinical features and monitoring in established disease.^135^ Screen-based eye-tracking (VOG) uses infrared light to track the reflection of the cornea and the centre of the pupil, allowing for the measurement of gaze directions and eye movements.

The combination of VR with eye-tracking and quantitative pupillometry technologies allows an immersive and controlled test environment and the delivery of precise, noninvasive, repeatable stimuli for objective oculometric assessment.^168,169^ This minimizes external distractions and variability in testing conditions, enhancing test reliability and accuracy.^168,169^ VR systems with integrated eye-tracking are designed for user-friendly operation by nonspecialist personnel and are preferred by patients over standard ocular motor screenings.^170^ This is important as access to neuro-ophthalmologists is limited.^171^

VR-based eye trackers can be useful in the clinical assessment of ocular motor and pupillary function, especially by capturing and quantifying subtle clinical signs that may go undetected with clinical assessments. These systems support the oculomics paradigm by enabling the objective quantification of ocular motor abnormalities that are often early indicators of neurological dysfunction, such as impaired saccades, fixation instability, abnormal smooth pursuit, and impaired vergence.^29,170,172-175^ Pupillary responses, which reflect autonomic function, can be captured, providing additional insights into conditions characterized by autonomic dysregulation and optic nerve damage.^60,176^ In a study, 239 people’s eye movements (114 PD patients, 125 healthy participants) were assessed using a VR headset with an embedded eye tracker connected to a computer to render four visual tasks.^173^ Based on the extracted ocular motor features, they built an AI-based model for PD diagnosis, obtaining an accuracy of 92.73%, and a receiver operator characteristic area under the curve (ROC-AUC) of 97.08%. This study highlights the diagnostic potential of VR-based oculometric tools.^173^ Other studies have corroborated the diagnostic and prognostic potential of eye-tracking and ocular motor measures to assess cognitive function and disease severity in PwMS.^38,45,49,51^ Studies also suggest that eye-tracking and pupillometric technologies can be used to monitor the effectiveness of treatments, such as dopaminergic therapy.^89,98,100^

The clinical application of VR-based eye-tracking technologies is still emerging. Future research should focus on validating these tools in larger, diverse populations. Integrating VR eye-tracking data with other diagnostic modalities, such as neuroimaging, electrophysiology, fluid biomarkers, and genetic testing, could further enhance diagnostic accuracy and understanding of disease mechanisms. To enable widespread clinical adoption, efforts should also be directed toward standardizing protocols and establishing normative datasets.

Despite these challenges, VR-based eye trackers represent a promising advancement in neuro-ophthalmology. As part of an oculomics-driven strategy, they have the potential to make advanced neurological assessments more accessible, objective, and efficient, facilitating earlier detection and improved management of neurological disorders.

## Conclusion

Ocular motor and pupillary biomarkers are gaining recognition as objective, quantifiable indicators of neurological dysfunction, offering significant potential for early diagnosis, disease monitoring, and treatment evaluation in neuroinflammatory and neurodegenerative disorders. Studies have shown that these biomarkers can capture subtle functional impairments in conditions such as MS, PD, and AD, often before structural changes become detectable.

The integration of ocular motor and pupillary biomarkers with structural and functional imaging, electrophysiology, as well as biochemical biomarkers within predictive models holds the potential to enhance early diagnostic accuracy, monitor disease progression, and evaluate treatment responses. Each condition presents distinct opportunities for combining these metrics into a multimodal biomarker framework.

As technology evolves, the integration of eye-tracking and quantitative pupillometry into wearable devices or VR-based platforms broadens the reach of oculometric assessments in clinical and research settings. These advancements may enable scalable and reproducible examination of ocular biomarkers, even in nonspecialist environments.

By refining and integrating these biomarkers into routine neurological evaluations, they may contribute to a paradigm shift in diagnostics, bridging the gap between research and real-world clinical application. Ultimately, oculomics and oculometrics hold promise for improving early detection, supporting differential diagnosis, and optimizing disease management across a wide range of neurological conditions.

However, to achieve widespread adoption, further large-scale validation studies, standardized protocols, and normative datasets are necessary to establish their clinical utility and ensure reliable implementation.

## Data Availability

No new datasets were generated or analysed for this review article. The case examples included in the manuscript were acquired using the neos™ medical system, and the underlying raw data and eye videos are not publicly available due to patient privacy regulations. The manuscript does not use custom code, and no analysis scripts were generated for this study.
